# Rapid Quality Identification of Decoction Pieces of Crude and Processed *Corydalis* Rhizoma by Near-Infrared Spectroscopy Coupled with Chemometrics

**DOI:** 10.1155/2021/1936057

**Published:** 2021-07-22

**Authors:** Weihao Zhu, Hao Hong, Zhihui Hong, Xianjie Kang, Weifeng Du, Weihong Ge, Changyu Li

**Affiliations:** ^1^College of Pharmaceutical Sciences, Zhejiang Chinese Medical University, Hangzhou 311402, China; ^2^Research Center of TCM Processing Technology, Zhejiang Chinese Medical University, Hangzhou 311401, China; ^3^Academy of Chinese Medical Sciences, Zhejiang Chinese Medical University, Hangzhou 311401, China

## Abstract

In order to identify the quality of crude and processed *Corydalis* Rhizoma decoction pieces, the research established a simple, fast, reliable, and validated near-infrared qualitative and quantitative model combined with chemometrics. 51 batches of crude and 40 batches of processed *Corydalis* Rhizoma from the Zhejiang and Jiangsu provinces of China were collected and analyzed. Crude and processed *Corydalis* Rhizoma samples were crushed to obtain NIR spectra. The content of seven alkaloids in crude and processed *Corydalis* Rhizoma was determined by high-performance liquid chromatography (HPLC). Pretreatment methods were screened such as normalization methods, offset filtering methods, and smoothing. Combined with partial least squares-discriminant analysis (PLS-DA) and partial least squares (PLS), the qualitative and quantitative models of crude and processed *Corydalis* Rhizoma were established, and the correlation coefficient (*R*^2^), root mean square error of calibration (RMSEC), and root mean square error of prediction (RMSEP) were used as evaluation indexes. Tetrahydropalmatine was used as an example for screening pretreatment methods; the results showed that MSC combined with the second derivative and no smoothing and the model with the wavelength range of 10000–5000 cm^−1^ had the best predictive ability and applied to all seven alkaloid components. Among them, the correlation coefficients were all higher than 0.99, and RMSEC and RMSEP were all less than 1%. The qualitative and quantitative model of the seven alkaloids in *Corydalis* Rhizoma can effectively identify the crude and processed *Corydalis* Rhizoma and determine the content of the seven alkaloids. By studying the NIR qualitative and quantitative models of crude and processed *Corydalis* Rhizoma, we can achieve rapid discrimination and quantitative prediction of crude and processed *Corydalis* Rhizoma. These methods can greatly improve the efficiency of traditional Chinese medicine analysis and provide a strong scientific basis for the quality identification and control of traditional Chinese medicine.

## 1. Introduction

*Corydalis* Rhizoma (referred as CR), named as Yuan Hu in Chinese, is the dried rhizome of *Corydalis yanhusuo* W. T. Wang [1]. Mainly produced in the Hebei, Shandong, Jiangsu, and Zhejiang provinces of China, CR has the effects of activating blood, moving Qi, and relieving pain in traditional Chinese medicine (TCM). In modern clinical applications, the stir-fried CR in vinegar is more widely used than the crude CR. Stir-frying with vinegar as a processing method of CR, in the process of stir-frying, the free alkaloid in CR is combined with acetic acid to form water-soluble acetate, which improves the frying rate [2]. According to the theory of TCM processing, the toxicity of drugs is reduced and the effects of moving Qi and relieving pain are enhanced by stir-frying with vinegar. Modern research showed that the seven free alkaloid compounds such as protopine, palmatine chloride, berberine, dehydrocorydaline, tetrahydropalmatine, tetrahydroberberine, and corydaline [3–9] in CR are the main active and characteristic components, which are often used to evaluate the quality of CR. Modern pharmacological studies have indicated that the alkaloid compounds in CR have sedative, analgesic, antitumor, and other pharmacological effects [10–15].

Currently, the processing level of the stir-fried CR in vinegar in the latest edition of the Pharmacopoeia of PRC is described as “the yellow-brown surface and cut surface with a slight vinegar aroma.” When the crude CR is left for a period of time, the cut surface will be black and similar in color to the processed CR. When the processed CR is left for a period of time, its vinegar smell will also be lost. Obviously, it is not enough to rely on the subjective judgment of the pharmacist by the surface color and aroma and it is difficult to ensure the overall quality of the processed CR. In addition, the Chinese Pharmacopoeia only stipulates the determination of the content of one alkaloid in CR, which cannot meet the quality control and requirements of CR before and after processing. According to reports in the literature, there are many methods of CR quality control before and after processing; for instance, high-performance liquid chromatography (HPLC) [16], ultra-performance liquid chromatography (UPLC) [17], thin-layer chromatography (TIC) [18], liquid chromatography-mass spectrometry (LC-MS), and gas chromatography-mass spectrometry (GC-MS) [19–22]. However, these methods have several disadvantages, such as cumbersome pretreatment, long time, high reagent consumption, and greater damage to the sample. Therefore, it is necessary to establish a fast and reliable method of CR quality control before and after processing in order to identify the quality of crude and processed CR quickly [23].

Near-infrared (NIR) spectroscopy, as a rapid identification analysis method that has the advantages of fast analysis speed, no damage to samples, and no pollution to the environment, has been widely used in many fields such as food, agriculture, and medicine. In recent years, the NIR spectroscopy technique has been successfully applied to the quality identification of *Salvia miltiorrhiza*, *Lonicerae japonicae flos*, *Scutellariae* radix, and other crude and processed medicinal materials [24–26]. At the same time, NIR spectroscopy has also been widely used in pharmaceuticals; for example, meningococcal polysaccharides, vardenafil tablets, and quinine drops [27–29]. Combined with chemometrics, NIR spectroscopy can quickly identify the quality of complex samples.

As far as we know, the quality identification of CR before and after processing by NIR spectroscopy combined with chemometrics has not been reported. In this study, protopine, palmatine chloride, berberine, dehydrocorydaline, tetrahydropalmatine, tetrahydroberberine, and corydaline were analyzed qualitatively and quantitatively by NIR spectroscopy combined with chemometrics [30]. It aims to establish a fast and reliable quality identification method. Finally, we established a qualitative and quantitative NIR model of crude and processed CR to achieve the qualitative discrimination of crude and processed CR, as well as the quantitative prediction of seven of the components, with accurate and reliable results.

## 2. Materials and Methods

### 2.1. Samples and Reagents

61 batches of crude CR and 50 batches of processed CR from the Zhejiang and Jiangsu provinces of China were collected and analyzed, and 10 batches from each class of the samples were selected as external validation samples. These medicinal materials have been identified by Professor Ping-fan Lai (Zhejiang Chinese Medical University). These samples were kept in the Research Center of Chinese Medicine Processing Technology of the Zhejiang University of Traditional Chinese Medicine. The reference of standards of protopine (99.6%, 110853-201805), palmatine chloride (85.7%, 110732-201913), berberine (86.7%, 110713-201814), and tetrahydropalmatine (99.9%, 110726-201819) were purchased from the National Institutes for Food and Drug Control (Beijing, China). Tetrahydroberberine (98%, 190144-201907) was purchased from Shanghai Hongying Biotechnology Co., Ltd (Shanghai, China). Dehydrocorydaline (95%, 30045-16-0) and corydaline (95%, 518-69-4) were purchased from Shanghai Standard Technology Co., Ltd (Shanghai, China). Acetonitrile (HPLC grade) and methanol (HPLC grade) were purchased from Tedia (Fairfield, USA). The purified water was purchased from Wahaha (Hangzhou Wahaha Group Co., Ltd., Hangzhou, China).

### 2.2. Standard and Sample Solution Preparation

All standard solutions for analysis were prepared in methanol. The concentrations of protopine, palmatine chloride, berberine, dehydrocorydaline, tetrahydropalmatine, tetrahydroberberine, and corydaline were 0.1045, 0.0992, 0.1022, 0.1036, 0.1075, 0.1215, and 0.1149 mg/ml, respectively. The standard solutions were filtered through a membrane filter (0.45 *μ*m) and preserved at 4°C.

The crude and processed CR powder (0.500 g, 50 mesh) was weighed and placed in a conical flask accurately. 50 ml of a concentrated ammonia solution-methanol (1 : 20) mixed solution was added and weighed accurately. The solution was cold-immersed for 1 h and then heated to reflux for 1 h, allowed to cool, and weighed again. The mixed solution of concentrated ammonia solution-methanol (1 : 20) was used to make up the lost weight, shaken, and filtered. The continuous filtrate was weighed 25 ml accurately and then evaporated filtrate to dryness. The residue was dissolved in methanol, transferred to a 5 ml volumetric flask, and diluted to the mark, then shaken, and filtered with a 0.45 *μ*m microporous filter membrane. The filtered continuous filtrate was used for HPLC quantitative analysis.

### 2.3. NIR Spectra Acquisition

Before the near-infrared spectroscopy analysis, the CR samples were crushed and passed through an 80-mesh sieve. NIR spectra of CR powder were acquired using a Thermo Antaris II Fourier transform spectrometer (Thermo Electron, USA) equipped with an integrating sphere, sample cup, and rotary tables [31, 32]. The result was analyzed by using TQ Analyst 8.3 software [33]. Each sample was taken 10 g, mixed evenly, and placed in a quartz sample cup, spread out, with the built-in background as a reference. The reference was subtracted, and then the spectrogram was collected. The sampling method is integrating sphere diffuse reflection, with wavenumber interval 4000–10000 cm^−1^, resolution 8.0 cm^−1^, scan signal accumulated 64 times, temperature (25 ± 2)°C, and relative humidity 45%–50%. Each sample was scanned three times, and the average spectrum was taken as the near-infrared spectrum of the sample.

### 2.4. HPLC Content Determination

Analyses were performed using the Thermo U3000 high-performance liquid chromatograph (Thermo Fisher Scientific) tandem diode array detector (DAD). An Agilent ZORBAX Extend-C_18_ (4.6 mm × 150 mm, 5 *μ*m) column was used at 25°C with a flow rate of 1.0 ml/min. The mobile phase was consisted of (A) 0.6% acetic acid aqueous solution (pH value adjusted to 5.0 by triethylamine) and (B) acetonitrile using a gradient elution of 17%–22% B (0–22 min), 22%–30% B (22–30 min), 30%–50% B (30–50 min), and 50%–80% B (50–75 min). The postequilibration time was 5 min.

### 2.5. NIR Spectral Data Pretreatment

The pretreatment methods of NIR spectra included the normalization methods, offset filtering methods, smoothing, and others. In normalization methods selection, when NIR diffuse reflectance spectra are collected, the optical path cannot be kept constant due to the influence of sample particle size and uniformity. In this case, multiple signal correction (MSC) or standard normal variate (SNV) is required to preprocess the spectra to eliminate these disturbances. In offset filtering methods selection, there are two processing methods of first derivative and second derivative, the purpose of which is to eliminate the baseline shift. In smoothing selection, there are three smoothing methods that can be used: no smoothing (NS), Savitzky–Golay filter (S-G), and Norris derivative filter (ND). Its purpose is to improve the signal-to-noise ratio, reduce random noise, and improve the stability of the model.

### 2.6. Establishment of Qualitative and Quantitative Models

The partial least squares (PLS) analysis is a multivariate data analysis method that combines regression modeling of multiple dependent variables on multiple independent variables and principal component analysis. It has the advantages of small calculation amount and high prediction accuracy, and it belongs to a bilinear model. The main idea is to linearly combine the independent variable and the dependent variable to convert them into new comprehensive variables that are independent of each other. Meanwhile, it is required to retain as much information of the original variable as possible and finally make a regression analysis. Partial least squares-discriminant analysis (PLS-DA) is a multivariate analysis method with supervised pattern recognition. It constructs a function that can judge the classification of unknown samples according to known classification criteria, thereby determining the attribution of the sample.

In this study, the PLS method in the near-infrared analysis software TQ Analyst (V9.8; Thermo Fisher Scientific, Waltham, MA, USA) was used to establish a quantitative model. First, we used correlation coefficient (*R*^2^), root mean square error of calibration (RMSEC), and root mean square error of prediction (RMSEP) as indicators to screen the optimal preprocessing method. Moreover, root mean square error cross-validation (RMSECV) and prediction residual error sum of squares (PRESS) were used as indicators to filter the optimal number of factors. Finally, the quantitative model was established for 91 batches of crude and processed CR samples (79 batches of calibration set, 12 batches of prediction set) with the optimal model pretreatment methods and number of factors, and *R*^2^, RMSEC, RMSEP, and RMSECV were used as model evaluation indicators to evaluate the near-infrared quantitative model of each component. At the same time, the established model was used to externally verify unknown samples. Similarly, we used the PLS-DA method to establish a qualitative model for crude and processed CR samples and performed PLS-DA on the near-infrared spectroscopy data of each batch of samples.

## 3. Results and Discussion

### 3.1. HPLC Analysis

**Figure 1:**
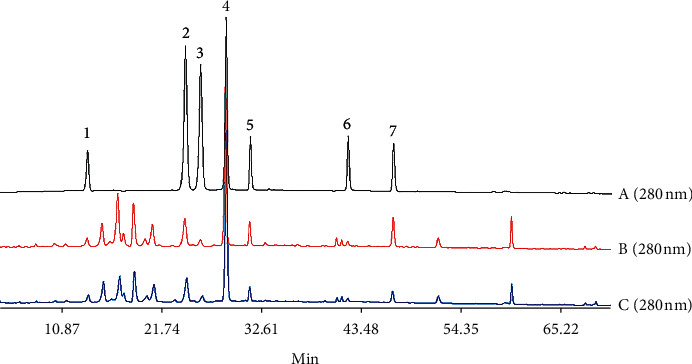
The HPLC chromatograms of mixed standards (a), crude CR (b), and processed CR (c). The peaks of 1, 2, 3, 4, 5, 6, and 7 represent protopine, palmatine chloride, berberine, dehydrocorydaline, tetrahydropalmatine, tetrahydroberberine, and corydaline, respectively.

An HPLC method for the rapid determination of alkaloids in decoction pieces of crude and processed CR was established and used for the analysis of all batches of samples. As shown in Figure 1, the retention time of the seven alkaloid components in the crude and processed CR extract solution was the same as the retention time of the standard solution. Meanwhile, these seven alkaloid components were separated well, and the content of them could be accurately determined by the external standard method. As illustrated in Table 1, the linear relationship was good and the RSD value of precision, stability, repeatability, and recovery were all less than 2%, proving that the method was suitable for quantitative analysis of all sample solutions.

### 3.2. NIR Spectra Analysis

#### 3.2.1. Selection of the Pretreatment Methods

The pretreatment methods were selected through correlation coefficient (*R*^2^), root mean square error of calibration (RMSEC), and root mean square error of prediction (RMSEP). The results of 7 components are illustrated in Tables 2–8. The results showed that in the comparison of different pretreatment methods, it was found that MSC combined with the second derivative and no smoothing method was proved to be the optimal pretreatment method.

**Figure 2:**
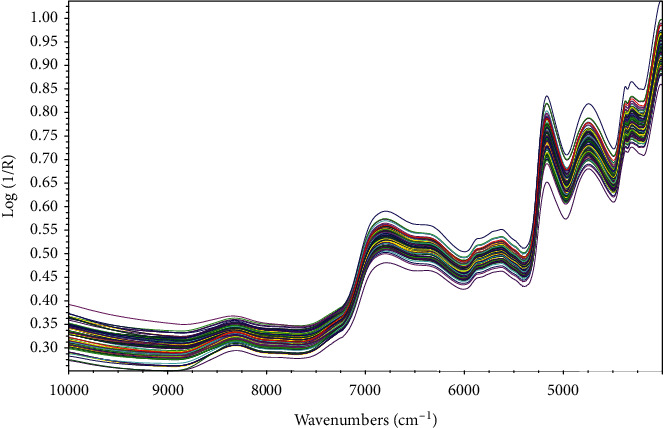
NIR spectra of 91 batches of crude and processed CR powder.

The original NIR average spectra from 10000 to 4000 cm^−1^ of 91 batches of crude and processed CR powder are shown in Figure 2. With the decrease of wavenumber, the absorption peak becomes stronger roughly and peaked at 4000 cm^−1^. Therefore, combined with the optimal pretreatment method after screening, the wavenumber range of 10000–5000 cm^−1^ with richer spectral information was selected to analyze the seven alkaloid components in crude and processed CR.

#### 3.2.2. Factor Selection

**Figure 3:**
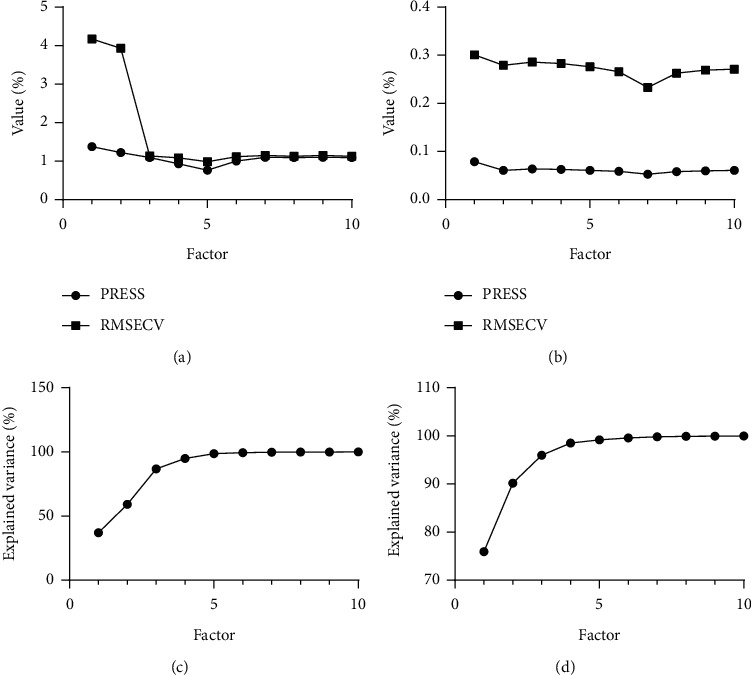
Correlation plots of factor, RMSECV, and PRESS: dehydrocorydaline (a) and tetrahydropalmatine (b). Correlation plots of the explained variance and factor: dehydrocorydaline (c) and tetrahydropalmatine (d).

When the PLS method is used to establish a quantitative model, the difference in the number of main factors led to a large difference in model prediction results. When the number of samples in the calibration set is determined, if the number of main factors is too large, noise will be introduced, resulting in the phenomenon of “overfitting.” If the number of main factors is too small, less spectral information will be used, resulting in poor model prediction ability. Root mean square error cross-validation (RMSECV) and prediction residual error sum of squares (PRESS) were used as indicators to investigate the influence of the number of main factors on the composition of 7 alkaloids, and the screening process is shown in Figures 3(a) and 3(b) by taking the example of dehydrocorydaline and tetrahydropalmatine. The correlation scatter plots of explained variance rate with factor changes are shown in Figures 3(c) and 3(d). The results are demonstrated in Table 9. When the number of main factors was 7, 6, 7, 5, 7, 7, and 5, the RMSECV value of the model was the lowest and the prediction accuracy of the model was better.

#### 3.2.3. Quantitative Model

12 batches of CR samples were randomly selected from 91 batches of crude and processed CR samples as the prediction set for evaluating the predictive ability of the model, and the remaining 79 batches were used as the calibration set for model establishment. This study used the partial least squares method combined with the best pretreatment method after screening to establish a quantitative model of seven alkaloids in crude and processed CR and evaluated the model with *R*^2^, RMSEC, and RMSEP simultaneously. The results are shown in Table 9.

**Figure 4:**
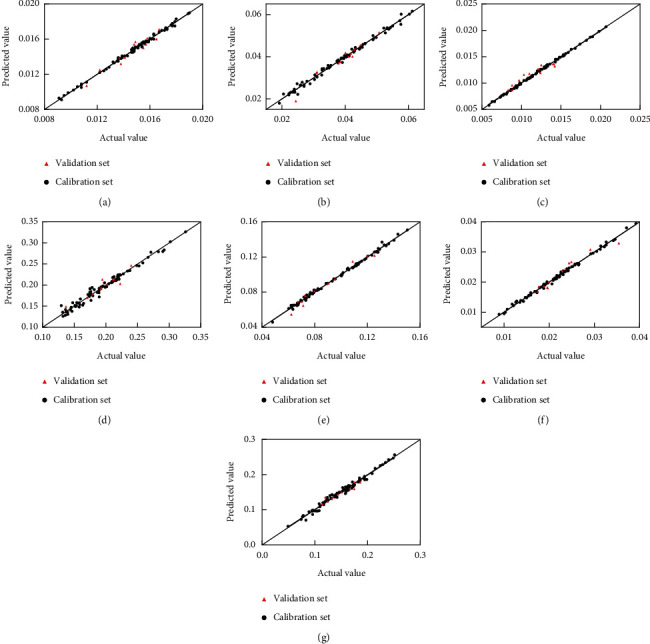
Correlation coefficient scatter plots of actual measured values and NIR predictions for protopine (a), palmatine chloride (b), berberine (c), dehydrocorydaline (d), tetrahydropalmatine (e), tetrahydroberberine (f), and corydaline (g).

Scatter plots of the actual measured values and predicted values of the seven alkaloid components modeled by using PLS combined with the pretreatment method are shown in Figure 4, and when the point was closer to the diagonal, the model's predictive performance was better. All the results in Table 9 and Figure 4 showed that the correlation coefficients were all higher than 0.99. RMSEC and RMSEP were all less than 1%, and the PLS combined pretreatment method model has good predictive performance when correlating the NIR spectra with the content of seven alkaloid components in crude and processed CR.

In order to analyze the accuracy of the model, the established models were used to analyze the unknown 20 batches of crude and processed CR (1 : 1). The results are indicated in Table 10. The correlation coefficients of actual value and predicted value were all greater than 0.99, which convincingly proved the stability of the model.

#### 3.2.4. Qualitative Model

In order to distinguish crude and processed CR, we established a qualitative model based on NIR spectra combined with chemometrics.

**Figure 5:**
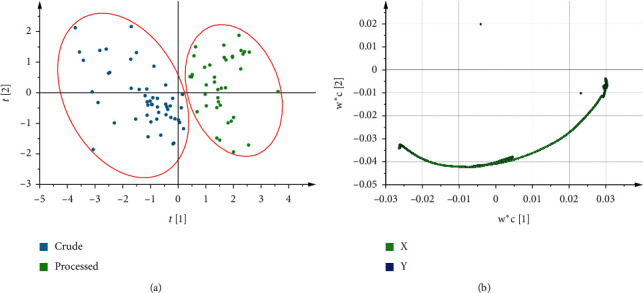
PLS-DA score scatter plot (a) and loading scatter plot (b) between crude and processed CR.

Similar to the quantitative model, we used PLS-discriminant analysis (DA) to classify crude and processed CR. The NIR spectra were imported to SIMCA-P (version 14.1; Umetrics AB, Umea, Sweden) for the PLS-DA analysis. The resulting score scatter plot is shown in Figure 5(a). It can be seen from Figure 5(a) that crude and processed CR were well distinguished, which proved that the qualitative model can be effectively used to identify crude and processed CR pieces. The loading scatter plot is shown in Figure 5(b). In Figure 5(b), the green point represented the *X*-variable (spectral data), and the blue point represented the *Y*-variable (the right represented the crude sample and the left point represented the processed sample). *X*-variables situated in the vicinity of the dummy *Y*-variables have the highest discriminatory power between the crude and processed CR.

**Figure 6:**
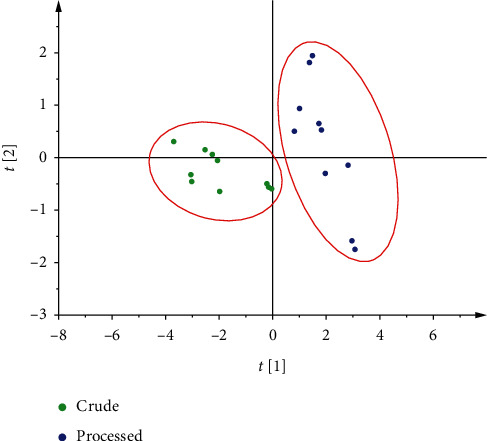
External validation score scatter plot in qualitative model.

In order to analyze the accuracy of the model, the established models were used to analyze the unknown 20 batches of crude and processed CR (1 : 1). The results are shown in Figure 6. The crude CR and processed CR were successfully identified by the qualitative model, which convincingly proved the stability of the model.

## 4. Conclusions

At present, CR is mainly studied by GC/MS for the analysis of crude and processed CR [19] or by HPLC fingerprinting combined with chemometric methods for its quality control [34], all of which have disadvantages such as destructive and low efficiency.

In this study, the qualitative and quantitative NIRS models of crude and processed CR were established to achieve rapid identification of crude and processed CR. First, the PLS-DA method was used to identify CR crude and processed samples successfully. In addition, we established a quantitative model by MSC combined with the second derivative and no smoothing pretreatment method, which can determine 7 components of alkaloids quickly and accurately. By studying the NIR qualitative and quantitative model of crude and processed CR, we can achieve rapid discrimination and quantitative prediction of crude and processed CR, and later, we will study the quality control of NIR in the whole process of CR. In the future, we hope that NIR can be applied to the quality control of CR and other TCMs. The overall results showed that the NIRS method was fast, simple, and efficient, which can greatly improve the efficiency of TCM analysis, and provided a strong scientific basis for the quality identification and control of TCM.

## Figures and Tables

**Table 1:** Methodological survey of HPLC results (*n* = 6).

Components	Standard curve	*R* ^2^	Linear range (*μ*g/ml)	Precision (RSD%)	Stability (RSD%)	Repeatability (RSD%)	Recovery	
							Mean	(RSD%)
Protopine	*Y* = 108.5*x* − 0.1987	0.9972	17.42–52.25	1.68	1.70	1.46	98.39	1.08
Palmatine chloride	*Y* = 531.89*x* − 0.6147	0.9981	16.53–49.60	1.21	1.56	1.53	96.77	1.95
Berberine	*Y* = 436.4*x* − 0.5199	0.9984	17.03–51.10	1.08	1.67	1.23	98.18	1.53
Dehydrocorydaline	*Y* = 395.47*x* − 0.1906	0.9990	17.27–51.80	0.53	1.15	1.14	97.91	1.74
Tetrahydropalmatine	*Y* = 109.5*x* − 0.0908	0.9991	17.92–53.75	0.24	0.78	1.35	101.62	1.29
Tetrahydroberberine	*Y* = 111.96*x* − 0.2056	0.9992	20.25–60.75	0.23	0.58	0.63	99.41	0.73
Corydaline	*Y* = 102.92*x* − 0.0599	0.9990	19.15–57.45	0.27	0.34	1.51	99.04	0.86

**Table 2:** Results with protopine of NIR spectra pretreatment methods.

Pretreatment	*R* ^2^	RMSEC (%)	RMSEP (%)
MSC + spectrum + NS	0.9433	1.2914	1.7347
MSC + 1stD + NS	0.9699	1.0277	1.4866
MSC + 2stD + NS	0.9978	0.3518	0.5573
MSC + spectrum + S-G	0.9419	1.4087	1.7568
MSC + 1stD + S-G	0.9666	1.0771	1.5615
MSC + 2stD + S-G	0.9228	1.6132	1.9441
MSC + 1stD + ND	0.9603	1.1747	1.5371
MSC + 2stD + ND	0.9548	1.2469	1.6983
SNV + spectrum + NS	0.9433	1.3982	1.7144
SNV + 1stD + NS	0.9699	1.0273	1.4836
SNV + 2stD + NS	0.9812	0.5576	0.7452
SNV + spectrum + S-G	0.9419	1.4028	1.7563
SNV + 1stD + S-G	0.9691	1.0328	1.5147
SNV + 2stD + S-G	0.9227	1.6133	1.4275
SNV + 1stD + ND	0.9606	1.1678	1.5336
SNV + 2stD + ND	0.9553	1.2466	1.6872

**Table 3:** Results with palmatine chloride of NIR spectra pretreatment methods.

Pretreatment	*R* ^2^	RMSEC (%)	RMSEP (%)
MSC + spectrum + NS	0.9451	1.3825	1.4038
MSC + 1stD + NS	0.9696	1.1018	1.3127
MSC + 2stD + NS	0.9959	0.4136	0.6458
MSC + spectrum + S-G	0.9438	1.3215	1.3924
MSC + 1stD + S-G	0.9697	1.1816	1.3327
MSC + 2stD + S-G	0.9027	1.8932	2.0041
MSC + 1stD + ND	0.9502	1.2400	1.4015
MSC + 2stD + ND	0.9569	1.2322	1.3898
SNV + spectrum + NS	0.9449	1.2538	1.3507
SNV + 1stD + NS	0.9694	1.1090	1.4316
SNV + 2stD + NS	0.9826	1.0015	1.0194
SNV + spectrum + S-G	0.9433	1.2375	1.3646
SNV + 1stD + S-G	0.9696	1.1083	1.3258
SNV + 2stD + S-G	0.9025	1.9032	2.0040
SNV + 1stD + ND	0.9498	1.3225	1.6090
SNV + 2stD + ND	0.9566	1.1237	1.3905

**Table 4:** Results with berberine of NIR spectra pretreatment methods.

Pretreatment	*R* ^2^	RMSEC (%)	RMSEP (%)
MSC + spectrum + NS	0.9523	1.1030	1.2841
MSC + 1stD + NS	0.9484	1.3150	1.6901
MSC + 2stD + NS	0.9994	0.0676	0.0764
MSC + spectrum + S-G	0.9507	1.0147	1.6187
MSC + 1stD + S-G	0.9499	1.3148	1.6515
MSC + 2stD + S-G	0.9430	1.3158	1.6913
MSC + 1stD + ND	0.8841	2.0221	2.1980
MSC + 2stD + ND	0.9644	1.0125	1.0160
SNV + spectrum + NS	0.9516	1.2450	1.4714
SNV + +1stD + NS	0.9463	1.3153	1.7031
SNV + 2stD + NS	0.9812	0.9651	0.0970
SNV + spectrum + S-G	0.9498	1.4081	1.7017
SNV + 1stD + S-G	0.9494	1.3481	1.6107
SNV + 2stD + S-G	0.9422	1.2857	1.6006
SNV + 1stD + ND	0.8840	2.0221	2.1970
SNV + 2stD + ND	0.9609	1.0131	1.0167

**Table 5:** Results with dehydrocorydaline of NIR spectra pretreatment methods.

Pretreatment	*R* ^2^	RMSEC (%)	RMSEP (%)
MSC + spectrum + NS	0.8949	1.9410	1.9641
MSC + 1stD + NS	0.9605	1.0088	1.0132
MSC + 2stD + NS	0.9872	0.6930	0.8810
MSC + spectrum + S-G	0.8900	1.9144	1.9166
MSC + 1stD + S-G	0.9698	1.0077	1.1204
MSC + 2stD + S-G	0.9692	1.0079	1.1205
MSC + 1stD + ND	0.9362	1.6111	1.7137
MSC + 2stD + ND	0.9451	1.4103	1.4021
SNV + spectrum + NS	0.8919	1.9143	1.9156
SNV + 1stD + NS	0.9607	1.0088	1.0131
SNV + 2stD + NS	0.9828	0.9878	0.9055
SNV + spectrum + S-G	0.8863	2.1460	2.1583
SNV + 1stD + S-G	0.9622	1.0085	1.3018
SNV + 2stD + S-G	0.9693	1.0078	1.2005
SNV + 1stD + ND	0.9362	1.6111	1.7136
SNV + 2stD + ND	0.9458	1.4102	1.5140

**Table 6:** Results with tetrahydropalmatine of NIR spectra pretreatment methods.

Pretreatment	*R* ^2^	RMSEC (%)	RMSEP (%)
MSC + spectrum + NS	0.9699	1.0060	1.0114
MSC + 1stD + NS	0.9092	1.7103	1.8104
MSC + 2stD + NS	0.9981	0.1594	0.3877
MSC + spectrum + S-G	0.9616	1.0068	1.0141
MSC + 1stD + S-G	0.8969	2.0110	2.0099
MSC + 2stD + S-G	0.7512	3.0164	3.0117
MSC + 1stD + ND	0.9081	1.8910	1.8003
MSC + 2stD + ND	0.8378	2.0536	2.8911
SNV + spectrum + NS	0.9836	0.8845	0.0935
SNV + 1stD + NS	0.9137	1.0101	1.0103
SNV + 2stD + NS	0.8887	2.0114	2.0111
SNV + spectrum + S-G	0.9610	1.0069	1.0162
SNV + 1stD + S-G	0.8631	2.4033	2.3503
SNV + 2stD + S-G	0.8724	2.1201	2.0012
SNV + 1stD + ND	0.8934	1.8112	1.9187
SNV + 2stD + ND	0.8724	2.6121	2.7184

**Table 7:** Results with tetrahydroberberine of NIR spectra pretreatment methods.

Pretreatment	*R* ^2^	RMSEC (%)	RMSEP (%)
MSC + spectrum + NS	0.9126	1.7854	018527
MSC + 1stD + NS	0.9677	1.0033	1.0037
MSC + 2stD + NS	0.9976	0.3920	0.6138
MSC + spectrum + S-G	0.9094	1.8555	1.8514
MSC + 1stD + S-G	0.9035	1.8457	1.8661
MSC + 2stD + S-G	0.9702	1.0632	1.0319
MSC + 1stD + ND	0.9228	1.8051	1.8043
MSC + 2stD + ND	0.9087	1.9055	1.8951
SNV + spectrum + NS	0.9172	1.8553	1.8543
SNV + 1stD + NS	0.9679	1.0313	1.0238
SNV + 2stD + NS	0.9760	1.0032	1.0024
SNV + spectrum + S-G	0.9138	1.7054	1.7054
SNV + 1stD + S-G	0.9033	1.9057	1.9061
SNV + 2stD + S-G	0.9704	1.0032	1.0039
SNV + 1stD + ND	0.9231	1.4051	1.4043
SNV + 2stD + ND	0.9087	1.9055	1.9052

**Table 8:** Results with corydaline of NIR spectra pretreatment methods.

Pretreatment	*R* ^2^	RMSEC (%)	RMSEP (%)
MSC + spectrum + NS	0.8614	2.5207	2.5157
MSC + 1stD + NS	0.9521	1.1125	1.1152
MSC + 2stD + NS	0.9918	0.5510	0.7230
MSC + spectrum + S-G	0.8599	2.6209	2.5798
MSC + 1stD + S-G	0.8928	2.0184	2.0168
MSC + 2stD + S-G	0.9534	1.1123	1.0153
MSC + 1stD + ND	0.8793	2.3950	2.3187
MSC + 2stD + ND	0.8368	2.7224	2.7701
SNV + spectrum + NS	0.8628	2.4207	2.4149
SNV + 1stD + NS	0.9521	1.2125	1.3153
SNV + 2stD + NS	0.9674	1.1089	1.1077
SNV + spectrum + S-G	0.7894	3.0251	3.0181
SNV + 1stD + S-G	0.8935	2.0183	2.0169
SNV + 2stD + S-G	0.9536	1.1230	1.1528
SNV + 1stD + ND	0.8795	2.3194	2.2887
SNV + 2stD + ND	0.8178	2.8935	2.8897

**Table 9:** Results of NIR quantitative model evaluation and selection of factors.

Components	*R* ^2^	RMSEC (%)	RMSEP (%)	RMSECV (%)	Factors
Protopine	0.9978	0.3518	0.5573	0.2220	7
Palmatine chloride	0.9959	0.4136	0.6458	0.7980	6
Berberine	0.9994	0.0676	0.0764	0.2540	7
Dehydrocorydaline	0.9872	0.6930	0.8810	0.9843	5
Tetrahydropalmatine	0.9981	0.1530	0.3810	0.2326	7
Tetrahydroberberine	0.9976	0.3920	0.6138	0.5480	7
Corydaline	0.9918	0.5510	0.7230	0.3385	5

**Table 10:** External validation in quantitative model.

Components	*R* ^2^	RMSEP (%)
Protopine	0.9921	0.1992
Palmatine chloride	0.9992	0.7564
Berberine	0.9986	0.1887
Dehydrocorydaline	0.9990	0.3481
Tetrahydropalmatine	0.9948	0.1416
Tetrahydroberberine	0.9931	0.2480
Corydaline	0.9995	0.1823

## Data Availability

The data used to support the results of this study are included within the article. Any further information is available from the authors upon request.
